# The interplay between spontaneous and controlled processing in creative cognition

**DOI:** 10.3389/fnhum.2014.00663

**Published:** 2014-08-28

**Authors:** Leh Woon Mok

**Affiliations:** Department of Psychology, College of Healthcare Sciences, James Cook UniversityTownsville, QLD, Australia

**Keywords:** creativity, spontaneous cognition, angular gyrus, lateral posterior parietal cortex, default-mode network, deliberate control, prefrontal cortex, multiple-demand network

## Abstract

Neural studies of creativity have yielded relatively little consistent results. For example, in functional neuroanatomical studies, the prefrontal cortex (PFC) has often been implicated as a critical neural substrate. However, results in electrophysiological (EEG) studies have been inconsistent as to the role of the PFC. EEG results have more often implicated widespread alpha synchronization, particularly in posterior regions, in creative cognition. Recent fMRI evidence has indicated that the PFC may be activated as a part of and together with other components of a deliberate control brain network. Controlled processing is neurologically dissociated from, but may co-occur with, spontaneous cognition mediated by a subset of the default-mode network (e.g., the angular gyrus [BA 39] in the posterior parietal cortex, which has been increasingly implicated in creative cognition). When the demand for controlled processing is substantially increased, default-mode processing may be suppressed. There is now preliminary evidence to suggest an association between alpha synchronization and default-mode processing. Creative cognition likely emerges from an *optimal* balance between spontaneous processing and controlled processing.

The advent of neuroimaging methodologies in the last two decades has seen an increase in studies of creativity from a cognitive neuroscience perspective. However, there is hitherto relatively little evidence for a clear and consistent pattern of brain activity that is associated with creative information processing in general, or with a particular proposed stage of it (see e.g., Arden et al., 2010; Dietrich and Kanso, 2010; Kaufman et al., 2010; Sawyer, 2011). Recent cognitive neuroscience evidence has supported the role that a subset of the brain’s default mode network (DMN) plays in spontaneous cognition (e.g., the angular gyrus, Brodmann Area [BA] 39, in the posterior parietal cortex; Mok, 2012). Default network activity may be suppressed when there is a substantial demand for controlled processing. However, spontaneous processing and controlled processing may co-occur. This article aims to highlight how these findings may be integrated with the existing creative cognition literature to offer a preliminary unifying perspective.

## Integration with existing themes

Neural studies of creativity have typically employed the standard, bipartite definition of creativity (Runco and Jaeger, 2012). Creativity is regarded as an ability that enables one to produce behavior or work that is *novel* and *useful*; useful creative behaviors and products would be appropriate and relevant to one’s social context (e.g., Amabile, 1982; Sternberg and Lubart, 1999). The focus is to illuminate the neurocognitive operations that occur during the creative *process* (Kaufman et al., 2010). In experimental studies of creativity, tests of divergent thinking have been widely used. It has been reasoned that cognitive processes that give rise to creative ideas are more likely generative and divergent in nature, where the thinker searches in various directions, leading to numerous varied possibilities and possibly novel combinations. Commonly used tests of divergent thinking include variants of the Alternate Uses Test (AUT), which requires the respondent to self-generate as many alternative or unusual uses for common objects as possible such as a paper clip or a brick; and the Torrance Tests of Creative Thinking (TTCT; Torrance, 1974), which is a battery of standardized verbal and figural tests. Despite a relative lack of consistent results, functional neuroanatomical studies using functional magnetic resonance imaging (fMRI) or positron emission tomography (PET) have often implicated the prefrontal cortex (PFC) as a critical neuroanatomical substrate of divergent thinking (e.g., Folley and Park, 2005; Goel and Vartanian, 2005; see Dietrich and Kanso, 2010). The PFC has long been thought to play a critical role in top-down cognitive control (Miller and Cohen, 2001). It has, thus, been deemed essential for common cognitive functions such as working memory, attentional control, cognitive flexibility and cognitive evaluation, all of which are thought to be important to creative information processing (Dietrich, 2004). However, when brain electrical activity was measured using electroencephalography (EEG), the divergent thinking paradigm did not yield comparable results in frontal regions (see e.g., Dietrich and Kanso, 2010).

In another line of cognitive neuroscience research, it has been demonstrated that the PFC, especially the dorsolateral aspect, may be activated as a part of and together with other components of (what is now known as) the multiple-demand network^1^ (Duncan, 2006). This form of cognitive processing is more deliberately engaged, attention-demanding, controlled and goal-directed, and is neurologically dissociated from, but may be concurrently experienced with, more spontaneous cognition mediated by a subset of the brain’s DMN (Mok, 2012). A default brain region that is particularly involved in spontaneous processing (e.g., spontaneous prospection) is the angular gyrus (BA 39) of the lateral posterior parietal cortex (LPPC), whether of events and experiences in the short-term (Mok, 2012) or the long-term (Andrews-Hana et al., 2010).

On a fundamental and individual level, real-life creative activities may be substantially facilitated by spontaneous cognition, mediated by a subset of the brain’s DMN and operating in the background as our concentration on the outside world is relaxed. However, when one is more focused on meeting more explicit and deliberate response demands such as in a test or timed situation, the demand for controlled processing may substantially increase, leading to a suppression of default processing (Mok, 2012). The differentiation between two processing modes—spontaneous vs. deliberate—for creative cognition has also been addressed in Dietrich (2004). More recent evidence is converging to indicate that creative cognition emerges from everyday cognitive abilities (Sawyer, 2011). Productive creative cognition may likely be facilitated by a delicate balance between more spontaneous processing vs. more controlled processing, both of which may be experienced concurrently to the extent that default activity does not become suppressed due to a substantial demand for controlled processing (Mok, 2012). In the literature, terms such as daydreaming and mind-wandering (e.g., Dietrich, 2007; Sawyer, 2011) have been used to describe mental states conducive to creative cognition. Such states are known to be mediated by the brain’s DMN. Default network activity increases when we are asleep or when we are undisturbed and left to ourselves to daydream and/or mind-wander (Mason et al., 2007; Buckner et al., 2008). Default-mode processing reflects the brain’s resting-state activity when our focus on the external environment is relaxed (Buckner et al., 2008). This corresponds to cognitive states described by disinhibition or relaxation of constraints (Dietrich, 2007; Kaufman et al., 2010) deemed to be facilitative of creativity.

Previously, in a PET study, BA 39 of the LPPC has been implicated in the performance of a verbal creativity task (Bechtereva et al., 2004). Participants generated a story using a difficult vs. an easy word list. The difficult list comprised remotely associated words, whereas the easy list comprised semantically-related words. Two other task conditions controlled for the syntactic vs. memory-related aspects of the task. When a task condition operationalized as requiring a greater degree of creativity to problem solve was contrasted against a task condition operationalized as requiring a lesser degree of creativity, at least a left BA 39 region within the LPPC (Cho, 2010) was implicated. A very recent fMRI study has also implicated the LPPC, including the angular gyrus, in creative cognition when participants generated alternate uses, as opposed to ordinary characteristics, for common objects (Kleibeuker et al., 2013). In a structural neuroanatomical MR study, a positive correlation has been found between cortical thickness in a right angular gyral region and self-reported real-life creative achievements (Jung et al., 2010).

The inconsistent pattern of results in neural studies of creativity has often been attributed to a lack of psychometrically sound assessment instruments and/or a definitional issue of the various concepts under investigation (e.g., Arden et al., 2010; Dietrich and Kanso, 2010). Below I outline two factors that may influence the observed results. A key aspect of the experimental tasks commonly used in neural studies of creativity such as those based on the AUT and the Torrance Tests is that they are administered in timed conditions, much like a test situation in which the participant may feel the stress of having their responses critically evaluated. Such external task demands may require more attention and control, leading to an increase in control network activity and a suppression of default network activity. Plucker and Makel (2010) have also raised concerns about a timed testing condition. Moreover, in an unpublished set of data, Mok and colleagues observed that the MR scanning environment such as the continuously loud scanner noise may lead to a decrease in size of experimental effects that are dependent on default network processing. To cope with the effect of environmental noise, there may be a greater demand for controlled processing, leading to a relative suppression of default network activity. These factors may contribute to why fMRI studies of creativity were in general rather consistent in implicating the PFC when timed tasks were used or when a study session felt like a test situation. Studies employing the EEG methodology with timed task administrations appeared to have yielded inconsistent results with regard to the role of frontal regions in creative cognition. The superior temporal resolution of the EEG methodology (1 ms, as opposed to 2 s for fMRI and 40 s for PET; Sawyer, 2011) may have afforded a greater potential to capture transient task-related activity such as default network activity, rather than possibly more persistent control network activity.

It is noteworthy that EEG studies have, in fact, been more consistent in showing that divergent thinking (measured by the AUT and/or the Torrance Tests) is associated with strong alpha activity and alpha synchronization, particularly in the parietal regions but also in the frontal and central regions (e.g., Fink et al., 2007, 2009a,b; Grabner et al., 2007). More recent studies employing simultaneous resting-state EEG/fMRI measurements have found a positive correlation between blood-oxygen-level dependent (BOLD) signals in components of the brain’s DMN, including the LPPC, and global alpha power (Mantini et al., 2007) and global alpha synchronization (Jann et al., 2009), respectively. Global measures of alpha activity were based on all electrode sites (frontal, central, temporal and posterior channels). Mantini et al. (2007) additionally found global alpha power to correlate negatively with an intrinsic connectivity network more involved in dorsal attention, which included the PFC. This line of research appears to provide preliminary support for an association between global alpha synchronization and a state of mind corresponding to the brain in default mode. In default mode, active processing in control, and related sensory and motor brain regions is depressed. This harks back to the traditional interpretation of alpha synchronization as reflective of “cortical idling” (Pfurtscheller et al., 1996). A modern interpretation of alpha synchronization may be that it is related to the brain’s default-mode processing.

Caution should be exercised in interpreting event-related alpha synchronization (ERS) as reflective of an inhibitory or top-down control process (cf. Klimesch et al., 2007). For example, alpha ERS observed over a short memory delay (1800 ms) in a working memory scanning task (a modified Sternberg task) has been interpreted as evidence against cortical idling but reflective of inhibition of brain areas not being used in processing (Schack and Klimesch, 2002). On close scrutiny, delay-period memory processing in this task might have been more spontaneously experienced than deliberately engaged, for the possibility that the probe stimulus, presented after the delay, would be contained in the (memory) set of simple stimuli presented before the delay. Delay-period memory processing of an (easy to discern) identity match stimulus may be facilitated, at default processing levels, concurrently by an LPPC region (for a prospective perspective) and a hippocampal region (for a retrospective perspective; Mok et al., 2009; Mok, 2012). When the memory set size was increased, delay-period ERS over posterior regions was stronger (Jensen et al., 2002; Schack and Klimesch, 2002). Upon presentation of the probe, event-related alpha desynchronization was observed (Klimesch et al., 2007). Participants likely engaged deliberately in controlled processing as they responded to the probe.

Alpha activity has also been taken as reflective of active inhibition of external sensory information during mental imagery (e.g., Cooper et al., 2003). More recent fMRI results, however, have supported the core, modality-independent role of the DMN in mental imagery (e.g., Mok et al., 2009; Daselaar et al., 2010). Another paradox was the simultaneous synchronization and desynchronization of different alpha responses in a recognition memory task (Klimesch et al., 2000). While transient evoked alpha synchronization may be observed at parieto-occipital sites, widespread induced alpha desynchronization may be observed at most recording sites. More spontaneously experienced cognitive processing in the LPPC, a DMN region, may co-occur with more controlled processing (Mok, 2012). The EEG signature of this co-occurrence warrants further investigation, as does a more systematic evaluation of the functional significance of alpha activity in general for spontaneous cognition mediated by a subset of the DMN.

## A preliminary unifying perspective and some definitional issues

As reviewed, the LPPC of the DMN is particularly involved in spontaneous cognition and it has increasingly been implicated in creative processing. Spontaneous processing in the LPPC is particularly of a prospective, or rather future-oriented, perspective. In the delay task used in Mok (2012), participants experienced spontaneous delay-period prospection of a cue-unique response outcome, mediated by the angular gyrus in the LPPC; there appeared to be individual differences in which hemisphere might be more involved. In accounting for semantic priming in cognitive psychology, the presentation of the prime stimulus that precedes the target stimulus has also been thought to initiate a prospective memory process, leading to spontaneous semantic spreading activation (Neely and Keefe, 1989). Divergent thought processes that underlie creative cognition, elicited by the initial stimulus of concern, may be quite similar in nature to spontaneous semantic spreading activation. Such thought processes allow one to access remote associates, which are important for the generation of novel possibilities and new associations (Kaufman et al., 2010). Functional neuroimaging studies have supported the role of the angular gyrus in a wide range of semantic tasks (e.g., Seghier et al., 2010). The subregion within the human angular gyrus most involved in spontaneous prospective memory processing at default activity level appears to be the posterior subdivision (PGp; Mok, 2012; Seghier, 2013).

The PFC activated as a part of a control network is well-poised to facilitate the evaluation and judgment of the social appropriateness of a novel idea, and the implementation of goal-directed plans (Dietrich, 2004). These processes will likely tap into contextual knowledge/memory whether social, cultural or historical. Depending on the content being processed, additional circuitries may be activated, e.g., sensory-perceptual cortices and related neural regions (for sensory-perceptual content such as visual and/or auditory content), emotional and associated limbic regions (for affective content), motivational systems (for appetitive or aversive content), and motor-related regions (for motor content). If additional neurocognitive analysis is involved, other relevant neural circuitries may be activated. Affective and/or motivational processes may modulate the balance between the deliberate and spontaneous processing modes and the efficacy of semantic associative processes (e.g., Shemyakina and Dan’ko, 2004), influencing the quality of ideational complexity vs. the effectiveness of judgment and implementation processes.

Runco (2010) introduced the notion of *optimization* and argued that it allows simplicity and parsimony in conceptualizing and defining creativity. Productive creative cognition likely emerges from an optimal balance between deliberate and spontaneous cognition. Runco (2010) further suggested that there is a single capacity, rather than two where one is for originality of thought and the other is for judiciousness, that underlies creative processing. On a neurocognitive level, in the service of everyday activities, this single capacity would correspond to the capacity to achieve, on a moment to moment basis, optimal balance between control network activity and default network activity, and optimal coordination between these respective networks and other brain regions involved in content processing. Evaluative and judgmental processes would be optimized to the extent of one’s understanding of the social, cultural and historical context. Morality and empathy, and how the social brain interacts with other components of the DMN (Mars et al., 2012) can potentially influence evaluative and judgmental processes and bear on eventual implementation of creative endeavors. The moment-to-moment optimization of spontaneous vs. controlled processing, whether the moment serves idea generation, verification or implementation (cf. Wallas, 1926), may be modulated by individual differences such as personality and intelligence. An empirical question is whether it would be more viable to define optimization relative to a self-norm or a population norm.

Novel ideas are rare, or rather deviant from the norm (Runco, 2004). Thought processes such as divergence and free association that give rise to deviant ideas are productively creative only to the extent that they are optimal but not excessive (Runco, 2004). The concept of deviance can be applied to accommodate documented links between creativity and deviant neurocognitive processes that underlie certain mental disorders (Kaufman et al., 2010), altered states of consciousness such as those induced by drugs, meditation, or long-duration exercise (Dietrich, 2007), and the “dark-side” of creativity such as the creation of weapons of mass destruction. Creativity studies with a developmental perspective (cf. Kleibeuker et al., 2013) should consider the developmental trajectory of the DMN vs. the control network and their respective connectivity with other brain regions; and the transition from relatively non-symbolic, language-free, and possibly innate abilities existing in infancy to symbolic or language-dependent abilities that may be modifiable and can benefit from training.

## Implications for future research and practice

To better measure default network activity, study sessions that feel like a test (e.g., with timed administrations) should be reconsidered. Study designs could bias participants toward different levels of creativity within-subject, carefully matching/controlling for as many of the other aspects of the experimental task as is possible. A concurrent-task design could allow for a better estimate of the relative levels of default network activity vs. control network activity. The potential influence of the MR scanning environment on default network suppression should also be systematically studied.

In the mainstream school system, the assessment of learning remains critical. A curricular emphasis on an end-product to be delivered by a certain deadline and its assessment as an outcome of learning may create a learning environment (akin to a test situation) that regularly heightens the brain’s control activity and suppresses default activity. Class activities should not engender persistent suppression of default processing on the individual level. Instead, with appropriate structuring and scheduling, more opportunities could be provided for developing minds and brains to spontaneously experience default-mode processing. Learning environments could be designed to enhance and/or maintain relevant default network activity despite existing external task demands. An appropriate use of prime stimuli to initiate optimal spontaneous semantic spreading activation could be a good starting point.

## Conflict of interest statement

The author declares that the research was conducted in the absence of any commercial or financial relationships that could be construed as a potential conflict of interest.

## Footnotes

^1^Components of the multiple-demand network include the pre-supplementary motor area, the inferior frontal sulcus and the lateral PFC around it [BA 9], the anterior insula and the intraparietal sulcus (Duncan, 2006). The multiple-demand network has been found to be activated in a wide range of cognitive activity, from working memory storage/manipulation, executive control to response selection (Woolgar et al., 2011).
